# Patient Factors Influencing the Prescribing of Lipid Lowering Drugs for Primary Prevention of Cardiovascular Disease in UK General Practice: A National Retrospective Cohort Study

**DOI:** 10.1371/journal.pone.0067611

**Published:** 2013-07-26

**Authors:** Jianhua Wu, Shihua Zhu, Guiqing Lily Yao, Mohammed A. Mohammed, Tom Marshall

**Affiliations:** 1 Centre for Environmental and Preventive Medicine, Barts and The London School of Medicine and Dentistry, University of London, London, United Kingdom; 2 School of Public Health and Population Science, University of Birmingham, Birmingham, United Kingdom; 3 Faculty of Medicine, University of Southampton, Southampton General Hospital, Southampton, United Kingdom; Iran University of Medical Sciences, Iran

## Abstract

**Background:**

Guidelines indicate eligibility for lipid lowering drugs, but it is not known to what extent GPs' follow guidelines in routine clinical practice or whether additional clinical factors systematically influence their prescribing decisions.

**Methods:**

A retrospective cohort analysis was undertaken using electronic primary care records from 421 UK general practices. At baseline (May 2008) patients were aged 30 to 74 years, free from cardiovascular disease and not taking lipid lowering drugs. The outcome was prescription of a lipid lowering drug within the next two years. The proportions of eligible and ineligible patients prescribed lipid lowering drugs were reported and multivariable logistic regression models were used to investigate associations between age, sex, cardiovascular risk factors and prescribing.

**Results:**

Of 365,718 patients with complete data, 13.8% (50,558) were prescribed lipid lowering drugs: 28.5% (21,101/74,137) of those eligible and 10.1% (29,457/291,581) of those ineligible. Only 41.7% (21,101/50,558) of those prescribed lipid lowering drugs were eligible. In multivariable analysis prescribing was most strongly associated with increasing age (OR for age ≥65 years 4.21; 95% CI 4.05–4.39); diabetes (OR 4.49; 95% CI 4.35–4.64); total cholesterol level ≥7 mmol/L (OR 2.20; 95% CI 2.12–2.29); and ≥4 blood pressure measurements in the past year (OR 4.24; 95% CI 4.06–4.42). The predictors were similar in eligible and ineligible patients.

**Conclusions:**

Most lipid lowering drugs for primary prevention are prescribed to ineligible patients. There is underuse of lipid lowering drugs in eligible patients.

## Introduction

Statins are known to be highly effective treatments for primary and secondary prevention of cardiovascular disease [1], [2], [3], [4]. Several guidelines have been issued at national and international level, recommending the use of statins in all patients who have a previous history of cardiovascular disease, or who are judged to be at high risk of developing cardiovascular disease [5], [6], [7], [8], [9], [10], [11], [12], [13]. UK guidelines set a treatment threshold of 20% ten-year CVD risk [8], [9], [11]. CVD risk is derived using a modified version of the Framingham risk equation [14], this requires information on age, gender, smoking status, diabetic status, systolic blood pressure, total cholesterol and high density lipoprotein (HDL) cholesterol levels. Risk is further adjusted for South Asian ethnicity and for family history of premature coronary heart disease [9], [11]. In addition, in UK guidelines diabetic patients aged over 40 years are considered eligible for lipid lowering therapy [8], [9], [10]. Patients with familial hypercholesterolaemia are eligible for lipid lowering drugs, irrespective of their calculated cardiovascular risk [8], [9], [15].

The use of calculated CVD risk as a criterion for recommending preventive drugs has a long history. CVD risk algorithms and equations have been available since the 1970s [16], [17], [18], [19], [20], [21]. The first equation from the Framingham cohort study was published and validated in 1976 [22], [23]. As early as 1978 it was demonstrated that multivariable risk predicted the benefits of preventive drugs [24]. Nevertheless early European, UK, US and Canadian lipid lowering guidelines recommended lipid lowering drugs if total cholesterol levels exceed a threshold, with some adjustment for the presence of categorical risk factors [25], [26], [27], [28]. Recognition that risk (and not cholesterol levels) predicted benefit was slow to gain acceptance and the concept of recommending treatment on the basis of CVD risk only emerged in the 1990s and later [29], [30], [31]. However, by 1998 UK guidelines clearly emphasised risk rather than individual risk factors as the basis for offering preventive drugs [32]. Current UK guidelines consistently recommended lipid lowering therapy for: patients whose calculated ten-year risk of CVD is ≥20%; diabetics aged ≥40 years; patients with familial hypercholesterolaemia [8], [9], [10], [11].

GP decision making may not have kept pace with changes in thinking around CVD prevention. In a secondary analysis of data from a UK CVD prevention project GP prescribing of statins in usual practice was associated with raised total cholesterol levels and with antihypertensive prescribing but not with other cardiovascular risk factors [33]. However in a subgroup of patients assessed by a cardiovascular prevention nurse, prescribing was associated with all the main cardiovascular risk factors and more consistent with guidelines. This analysis raised questions about the patient factors associated with statin prescribing. In the absence of advice from a specialist nurse, GP prescribing behaviour systematically diverged from current guidelines: more closely associated with categorical clinical characteristics than calculated risk. This behaviour is more consistent with previous than current CVD prevention guidelines. Understanding which clinical characteristics are associated with prescribing therefore provides insight into GPs understanding of prevention.

However it is unclear to what extent the findings of this study apply to other settings as it was confined to six general practices in a single urban area in the West Midlands in the context of a specific cardiovascular prevention project.

This present study uses a large dataset of electronic primary care records from general practices across the UK. It aims to investigate the prescribing of lipid lowering drugs for patients without existing cardiovascular disease in relation to their eligibility under clinical guidelines. We then investigate the association between patient characteristics and GP prescribing of lipid lowering drugs in patients without existing cardiovascular disease.

## Methods

### Data sources

This is a retrospective cohort study using data from a database of electronic primary care records: The Health Improvement Network (THIN). Data are uploaded from UK general practices that use the VISION computer system and used for research [34]. The National Health Service (NHS) South-East Multi-centre Research Ethics Committee approved THIN data collection in 2003. Under the terms of this approval the data are anonymised so that neither individual patients nor individual general practices can be identified; because of this individual patient consent is not required analysis of the dataset (nor is it possible since individuals cannot be identified); studies using pre-collected, anonymised data must undergo scientific review to ensure appropriate analysis [35]. For this study, further scientific review and ethical approval was obtained by TM from the National Research Ethics Service for the NHS (reference 08/H0305/3).

More than 5 million anonymised patients are collected from 421 practices that are broadly representative of UK general practice in terms of patients' age and sex, practice size, and geographical distribution. The database includes coded data on all diagnoses, consultations, prescriptions, measurements and laboratory investigations. The analysis included all patients aged between 30 and 74 in the database on 1^st^ May 2008 (the index date) who were not currently receiving a prescription for a lipid lowering drug and provided they had at least one year of records prior to the index date. To avoid the problem of inflation of the denominator population during periods when deaths are under-recorded, practice records were excluded if they fell before a period of acceptable mortality recording [36]. Because the database was extracted on 1^st^ May 2010 this meant that patients had up to two years of follow up from the index date.

### Exclusions

**Figure 1:**
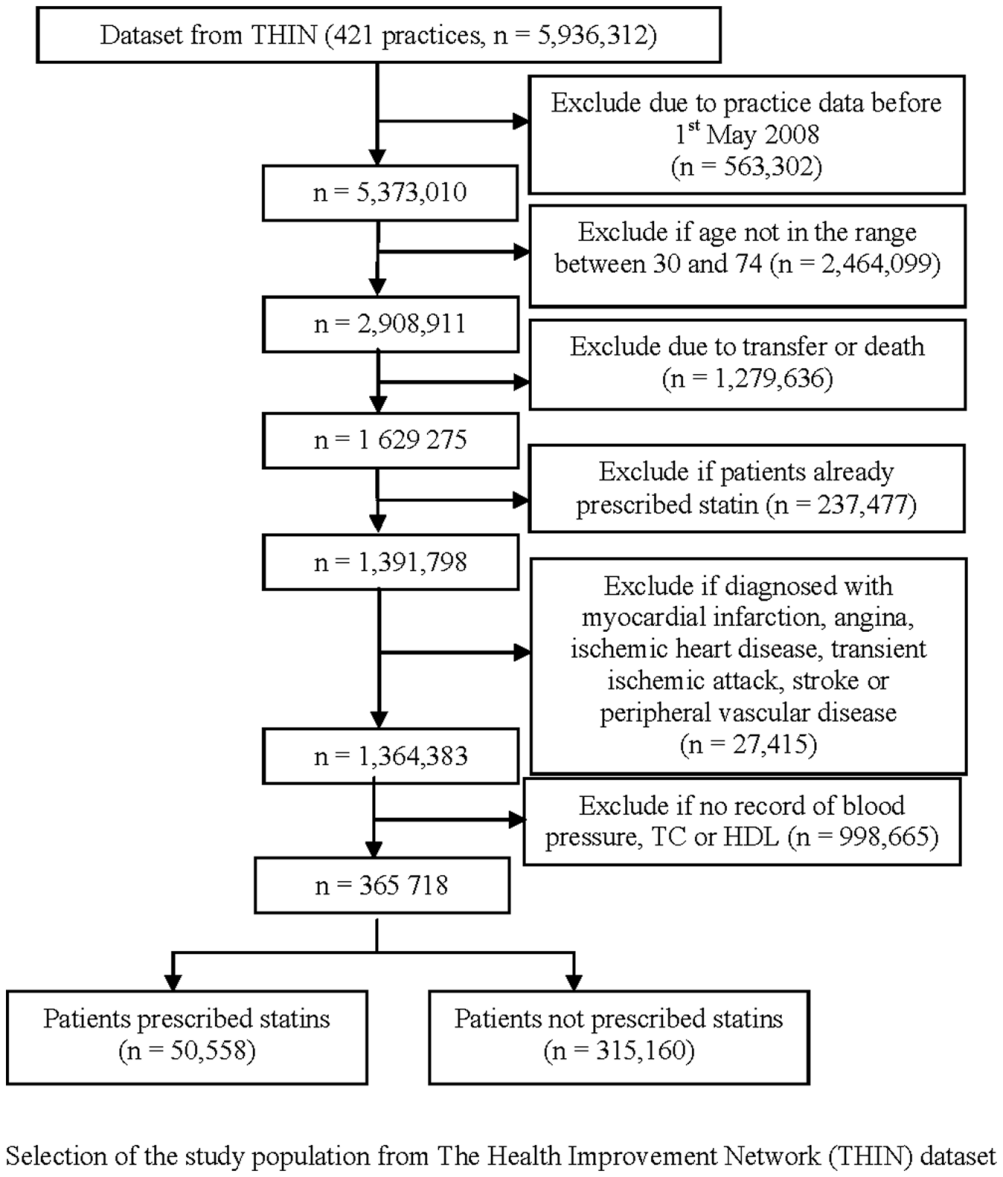
Flow of patients through study.

Only patients without clinical evidence of CVD (myocardial infarction, ischaemic heart disease, angina, transient ischemic attack and stroke) before the date of prescribing a lipid lowering drug or the last date of follow up were included in the analyses (Figure 1). All patients with CVD are eligible for lipid lowering treatment [8], [9], [11]. Factors influencing prescription of lipid lowering treatment may be different in these patients and it is not possible to calculate their ten-year cardiovascular risk. They were therefore excluded and will be the subject of a separate analysis.

### Predictor variables and outcome

Patients' age (years), sex, practice and quintile of area deprivation score (Townsend score) [37] based on their residential postcode were extracted from the dataset. In the database, medical diagnoses are coded using the Read code classification scheme [38]. An appropriate diagnostic Read code at any time before the index date was taken to indicate the presence of the diagnosis at baseline. The use of antihypertensive drugs was also recorded.

For measurement variables - systolic and diastolic blood pressure (mm Hg), serum total cholesterol (TC, mmol/L), HDL cholesterol (HDL, mmol/L) - the most recent records during the period of follow up were used. These could be up to one year prior to the index date or the final value during the two years of follow up. We excluded implausible values of the measurement variables. The most recent smoking status (non-smoker, ex-smoker and current smoker) was also included. If no record existed for smoking in that period, the patient was assumed to be non-smoker. Previous analyses suggest that the prevalence of smoking in the THIN database is similar to that seen in other sources and that those with missing smoking status are likely to be ex-smokers or non-smokers [39]. Full lists of Read codes used are available from the authors.

Our dataset did not include all GP contacts but we determined the number of blood pressure recordings carried out in the last year of follow up and used this as proxy for frequency of GP visits.

Although we are primarily interested in statin prescribing we used first prescription of a lipid lowering drug at any point during the period of follow up as the outcome. This is because GPs may prescribe another lipid lowering agent instead and are unlikely to prescribe both. In practice this makes little difference as in England 93% of prescriptions for lipid lowering drugs are for statins [40].

### Primary analysis and sensitivity analysis including missing values

Calculating ten-year risk requires knowledge of age, sex, diabetic status, smoking status, total cholesterol, HDL cholesterol and systolic blood pressure. Our primary analysis made use of cases for whom these data were complete. We undertook a sensitivity analysis using all patients including those with missing data. Since the absence of lipid levels and blood pressures may be related to the prescription of a lipid lowering drug, for each of the missing continuous variables we created a “missing value” category.

### Determining cardiovascular risk

Ten-year cardiovascular risk was calculated from each patient's risk factors using the modified Framingham equation advocated in 2005 UK guidelines [8]. This method calculates cardiovascular risk as the sum of coronary heart disease risk and stroke risk, multiplying by 1.5 for family history of premature coronary heart disease.

### Treatment eligibility

For each patient, treatment eligibility was determined from their cardiovascular risk, diabetic status, total cholesterol and HDL cholesterol levels.

For this analysis, patients were considered to be eligible for lipid lowering drugs they met the relevant criteria in the principal relevant UK guidelines. This includes Scottish and joint British guidelines on cardiovascular prevention, NICE guidelines on diabetes, NICE guidelines on familial hyperlipidaemia and NICE guidelines on lipid lowering [8], [9], [10], [11], [15]. Patients were eligible for treatment if their ten-year CVD risk was ≥20% [8], [9], [11] or if they were diabetic and aged ≥40 years [8], [9], [10]. In addition, patients were considered eligible for lipid lowering drugs if they had familial hypercholesterolaemia [8], [9], [10], [11], [15]. Familial hypercholesterolaemia is poorly coded in electronic primary care records. We identified patients aged 30 years to 39 years with a total cholesterol ≥8.8 mmol/L and aged ≥40 years with a total cholesterol ≥9.3 mmol/L this has a specificity of 0.98 for a diagnosis of familial hypercholesterolaemia [41]. This definition may overestimate the prevalence of hypercholesterolaemia, as the condition affects 1 in 500 of the population [42]. One guideline also consider all patients whose total cholesterol to HDL cholesterol ratio is ≥6 to be eligible for lipid lowering drugs, therefore these were also considered eligible for treatment [8].

### Statistical Analyses

We first describe prescribing in relation to eligibility under UK clinical guidelines. For subsequent analyses continuous variables were categorised. Age (years) was categorised into four bands (≤44, 45–54, 55–64 and ≥65). Total cholesterol was divided into three categories (<5, 5.0–6.9 and ≥7 mmol/L) as these thresholds are used in some local clinical guidelines. Systolic and diastolic blood pressure were divided into three categories (<140, 140–159 and ≥160 mm Hg) and (<90, 90–99 and ≥100 mm Hg). As there are no clinically accepted thresholds for HDL cholesterol (mmol/L) this was divided into quartiles (≤1.2, 1.3–1.4, 1.5–1.7 and ≥1.8 mmol/L). The frequency of blood pressure measurements in the past year was also categorized into 3 levels (0, 1–3 and ≥4).

In the multivariable logistic model, we included all risk factors. We also carried out stratified analyses for patients eligible and not eligible for lipid lowering drugs under UK guidelines in order to investigate the relationship between predictors in the model and patient eligibility.

We used robust standard errors throughout to account for dependency between patients clustered within the same practice. We also undertook secondary analyses to examine the role of variation between practices, because some practices may differ in their overall propensity to prescribe lipid lowering drugs. We performed all analyses using multilevel random intercept logistic regression models with patients nested in practices and with robust standard errors.

To determine whether cardiovascular risk factors might have an additional influence on prescribing beyond their contribution to the cardiovascular risk equation, further analysis was undertaken including ten year cardiovascular risk as well as individual risk factors.

For the sensitivity analysis using complete cases, an additional “missing” category was created for total cholesterol, HDL cholesterol, systolic and diastolic blood pressure. It is not possible to calculate CVD risk for patients with missing blood pressures or cholesterol measurements, however patients with missing risk factor data may be identified as eligible for treatment if they are diabetic and aged ≥40 years and (if cholesterol levels are available) they have familial hypercholesterolaemia.

All analyses were performed using SAS version 9.2 for windows (SAS Institute Inc., Cary, NC).

## Results

There were 1,364,383 patients without clinical evidence of cardiovascular disease who were not on lipid lowering drugs at baseline, after exclusion of those without records of blood pressure, total cholesterol or HDL there were 365,718 complete cases for analysis (Figure 1). Table 1 shows the characteristics of the study population divided into those eligible and ineligible for lipid lowering drugs. Overall 6.7% of this untreated cohort was diabetic, 6.4% had a family history of premature CHD, 16.3% were current smokers and 99.9% had a record of their smoking status. In total, 13.8% (50,558/365,718) were prescribed lipid lowering drugs by the end of the two years of follow up: 28.5% (21,101/74,137) of those eligible under UK guidelines and 10.1% (29,457/291,581) of those ineligible. Therefore 41.7% (21,101/50,558) of those prescribed lipid lowering drugs were eligible.

**Table 1:** Characteristics of the study population subgrouped into patients eligible and ineligible for lipid lowering drugs.

	Number (%) of patients in each category taking lipid lowering drugs			
	Eligible patients		Not eligible patients	
Characteristics	No lipid lowering drugs (n = 53,036)	Lipid lowering drugs (n = 21,101)	No lipid lowering drugs (n = 262,124)	Lipid lowering drugs (n = 29,457)
Men	41,211 (77.7)	14,980 (71.0)	99,622 (38.1)	13,377 (45.4)
Women	11,825 (22.3)	6,121 (29.0)	162,502 (61.9)	16,080 (54.6)
Age group:				
≤44	7,730 (14.6)	1,777 (8.4)	63,484 (24.2)	2,447 (8.3)
45–54	11,476 (21.6)	4,770 (22.6)	82,536 (31.5)	6,308 (21.4)
55–64	16,470 (31.1)	7,682 (36.4)	74,703 (28.5)	11,674 (39.6)
≥65	17,360 (32.7)	6,872 (32.6)	41,401 (15.8)	9,028 (30.7)
Diabetes:				
Yes	12,129 (22.9)	10,030 (47.5)	1,608 (0.6)	555 (1.9)
No	40,907 (77.1)	11,071 (52.5)	260,516 (99.4)	28,902 (98.1)
Family history of CHD:				
Yes	4,710 (8.9)	1,897 (9.0)	14,796 (5.6)	2,040 (6.9)
No	48,326 (91.1)	19,204 (91.0)	247,328 (94.4)	27,417 (93.1)
Smoking status:				
Non-smoker	16,564 (31.2)	6,452 (30.6)	147,839 (56.4)	14,371 (48.8)
Current smoker	10,507 (19.8)	4,687 (22.2)	38,535 (14.7)	5,790 (19.7)
Former smoker	25,912 (48.9)	9,958 (47.2)	75,409 (28.8)	9,278 (31.5)
Missing data	53 (0.1)	4 (0)	341 (0.1)	18 (0.1)
Total cholesterol (mmol/L): mean (SD)	5.6 (1.2)	5.4 (1.4)	5.4 (0.9)	5.3 (1.2)
HDL cholesterol (mmol/L): mean (SD)	1.1 (0.3)	1.2 (0.3)	1.2 (0.3)	1.5 (0.6)
Systolic BP (mm Hg): mean (SD)	139.2 (15.7)	140.0 (14.8)	132.3 (15.2)	137.3 (14.2)
Diastolic BP (mm Hg): mean (SD)	82.1 (9.2)	82.0 (8.8)	80.3 (9.3)	81.2 (8.5)
Frequency of BP measurements During last year of follow up:				
0	11,873 (22.4)	1,183 (5.6)	78,737 (30.0)	3,084 (10.5)
1–3	32,828 (61.9)	13,799 (65.4)	154,698 (59.0)	19,127 (64.9)
≥4	8,335 (15.7)	6,119 (29.0)	28,689 (10.9)	7,246 (24.6)
CVD risk: mean (SD)	20.5% (8.6%)	21.1% (8.7%)	7.4% (5.0%)	10.4% (4.8%)

*Note: for total cholesterol, HDL cholesterol, systolic blood pressure, diastolic blood pressure, the mean and standard deviation (SD) are presented for each category.*

In univariable analysis increasing age, diabetic status, prescription of antihypertensive drugs, frequent blood pressure measurements and eligibility for lipid lowering drugs were all strong predictors of treatment. Eligible patients were more likely to be treated than those not eligible. But eligibility because of diabetes in patients age ≥40 years or familial hypercholesterolaemia were much stronger predictors of treatment than eligibility because of a ten-year CVD risk ≥20% or a total cholesterol to HDL cholesterol ratio ≥6.0. (Table 2)

**Table 2:** Univariable analysis of factors associated with prescribing lipid lowering drugs.

Univariable models	Patients (n = 365,718)		
	OR	95% CI	P value
Age group:			
≤44	1		<0.001
45–54	2.02	1.95–2.10	
55–64	3.76	3.63–3.89	
≥65	4.87	4.70–5.05	
Gender (Men vs Women)	1.59	1.56–1.62	<0.001
Smoking status*	1.54	1.51–1.57	<0.001
Diabetes*	6.04	5.87–6.21	<0.001
Family history of CHD*	1.27	1.23–1.32	<0.001
Antihypertensive drugs use*	2.65	2.60–2.70	<0.001
Deprivation (Townsend fifth):			
1 (least deprived)	1		<0.001
2	1.10	1.07–1.13	
3	1.09	1.06–1.12	
4	1.21	1.18–1.25	
5 (most deprived)	1.30	1.25–1.34	
Total Cholesterol (mmol/L):			
<5.0	1		<0.001
5.0–6.9	0.61	0.59–0.62	
≥7.0	1.36	1.32–1.41	
HDL Cholesterol (mmol/L):			
≤1.2	1		<0.001
1.3–1.4	0.78	0.76–0.80	
1.5–1.7	0.63	0.61–0.65	
≥1.8	0.48	0.47–0.49	
Systolic blood pressure (mm Hg):			
<140	1		<0.001
140–159	1.55	1.52–1.59	
≥160	1.74	1.68–1.81	
Diastolic blood pressure (mm Hg):			
<90	1		<0.001
90–99	0.92	0.89–0.95	
≥100	0.86	0.79–0.93	
Frequency of BP measurements:			
0	1		<0.001
1–3	3.73	3.61–3.85	
≥4	7.67	7.39–7.95	
**Eligibility for lipid lowering drugs**			
Familial Hypercholesterolaemia	5.38	4.33–6.67	<0.001
TC/HDL≥6	1.83	1.77–1.89	<0.001
Diabetic≥40 years	6.44	6.26–6.63	<0.001
Ten year CVD risk ≥20%	3.01	2.94–3.09	<0.001
All eligible	3.64	3.56–3.71	<0.001

*OR = odds ratio, CI = confidence interval.*

*Adjusted for clustering by practice.*

* *Referent is none or no disease.*

In multivariable analysis including individual risk factors the likelihood of a prescription of lipid lowering drugs was most strongly associated with increasing age (Odds Ratio for age ≥65 years 4.21; 95% CI 4.05–4.39); diabetes (Odds Ratio 4.49; 95% CI 4.35–4.64); total cholesterol level (Odds Ratio for total cholesterol ≥7 mmol/L 2.20; 95% CI 2.12–2.29); and the frequency of blood pressure measurements in the past year (Odds Ratio for ≥4 measurements 4.24; 95% CI 4.06–4.42). (Table 3) Other characteristics, such as male sex, receiving a prescription for antihypertensive drugs, smoking status and family history of premature coronary heart disease were moderately associated with prescribing lipid lowering drugs. There was a modest trend towards increased prescribing to patients in more deprived areas.

**Table 3:** Multivariable odds ratios for association between individual patient risk factors and prescription of lipid lowering drugs.

Multivariable models*	Patients (n = 365,718)		
	OR	95% CI	P value
Age group:			
≤44	1		<0.001
45–54	2.01	1.93–2.09	
55–64	3.59	3.45–3.73	
≥65	4.21	4.05–4.39	
Gender (Men vs Women)	1.46	1.42–1.49	<0.001
Smoking status**	1.35	1.32–1.38	<0.001
Diabetes**	4.49	4.35–4.64	<0.001
Family history of CHD**	1.52	1.46–1.59	<0.001
Antihypertensive drugs use**	1.46	1.43–1.50	<0.001
Deprivation (Townsend fifth):			
1 (least deprived)	1		<0.001
2	1.03	1.00–1.06	
3	1.03	0.99–1.06	
4	1.09	1.05–1.12	
5 (most deprived)	1.15	1.10–1.20	
Total Cholesterol (mmol/L):			
<5.0	1		<0.001
5.0–6.9	0.81	0.79–0.83	
≥7.0	2.20	2.12–2.29	
HDL Cholesterol (mmol/L):			
≤1.2	1		<0.001
1.3–1.4	0.87	0.84–0.89	
1.5–1.7	0.74	0.72–0.76	
≥1.8	0.57	0.56–0.60	
Systolic blood pressure (mm Hg):			
<140	1		<0.001
140–159	1.09	1.07–1.12	
≥160	1.07	1.02–1.12	
Diastolic blood pressure (mm Hg):			
<90	1		<0.001
90–99	0.93	0.90–0.96	
≥100	0.85	0.79–0.91	
Frequency of BP measurements:			
0	1		<0.001
1–3	2.61	2.51–2.70	
≥4	4.24	4.06–4.42	

*Adjusted for clustering by practice.*

* *Each risk factor is independently adjusted for other risk factors.*

** *Referent is none or no disease.*

Multivariable analyses were also performed separately on subgroup patients who were eligible and ineligible for lipid lowering drugs (Table 4). Predictors of treatment were very similar in eligible and ineligible patients but some characteristics were more strongly associated with prescribing among ineligible patients. Among eligible patients the odds ratio for diabetes was 3.41 (95% CI 3.25 to 3.58) and for age ≥65 years 1.73 (95% CI 1.62 to 2.85). Among ineligible patients the odds ratio for diabetes was 9.10 (95% CI 8.13 to 10.10) and for age ≥65 years 6.74 (95% CI 6.42 to 7.08). Among ineligible patients male sex, receiving a prescription for antihypertensive drugs, smoking status and family history of premature coronary heart disease were slightly more strongly associated with prescribing lipid lowering drugs. A trend towards increased prescribing to patients from more deprived areas was present in both eligible and ineligible patients.

**Table 4:** Multivariable odds ratios for association between individual risk factors and lipid lowering drugs in eligible and ineligible patients.

Multivariable model*	Eligible patients (n = 74,137)			Ineligible patients (n = 291,581)		
	OR	95% CI	P value	OR	95% CI	P value
Age group:						
≤44	1		<0.001	1		<0.001
45–54	1.58	1.48–1.70		2.51	2.38–2.65	
55–64	1.98	1.86–2.12		5.28	5.00–5.57	
≥65	1.75	1.63–1.88		7.41	7.00–7.84	
Gender (Men vs Women)	1.13	1.08–1.18	<0.001	1.59	1.55–1.63	<0.001
Smoking status**	1.26	1.21–1.31	<0.001	1.42	1.38–1.45	<0.001
Diabetes**	3.43	3.27–3.59	<0.001	8.86	7.90–9.94	<0.001
Family history of CHD**	1.22	1.14–1.30	<0.001	1.70	1.61–1.80	<0.001
Antihypertensive drugs use**	1.26	1.21–1.31	<0.001	1.61	1.57–1.66	<0.001
Deprivation (Townsend fifth):						
1 (least deprived)	1		<0.001	1		0.002
2	1.06	1.01–1.12		1.02	0.98–1.06	
3	1.05	0.99–1.11		1.01	0.97–1.05	
4	1.12	1.06–1.19		1.06	1.01–1.11	
5 (most deprived)	1.19	1.11–1.27		1.10	1.04–1.16	
Total Cholesterol (mmol/L):						
<5.0	1		<0.001	1		<0.001
5.0–6.9	1.09	1.05–1.14		0.65	0.65–0.70	
≥7.0	2.48	2.32–2.66		1.61	1.53–1.70	
HDL Cholesterol (mmol/L):						
≤1.2	1		<0.001	1		<0.001
1.3–1.4	0.99	0.95–1.04		0.91	0.87–0.94	
1.5–1.7	0.93	0.88–0.98		0.77	0.74–0.81	
≥1.8	0.85	0.79–0.92		0.61	0.58–0.64	
Systolic blood pressure (mm Hg):						
<140	1		0.05	1		<0.001
140–159	1.02	0.98–1.07		1.12	1.09–1.16	
≥160	0.94	0.88–1.01		1.14	1.07–1.22	
Diastolic blood pressure (mm Hg):						
<90	1		0.002	1		<0.001
90–99	0.94	0.89–0.99		0.91	0.87–0.95	
≥100	0.84	0.75–0.94		0.81	0.73–0.89	
Frequency of BP measurements:						
0	1		<0.001	1		<0.001
1–3	3.15	2.95–3.37		2.35	2.25–2.45	
≥4	4.96	4.60–5.37		3.88	3.69–4.09	

*Adjusted for clustering by practice.*

* *Each risk factor is independently adjusted for other risk factors.*

** *Referent is none or no disease.*

Univariable analysis showed a linear relationship between cardiovascular risk and prescribing lipid lowering drugs, with no threshold at 20% ten-year CVD risk. (Table 5) Adding 10-year cardiovascular risk to the individual risk factors model made little difference to the odds ratios (data not shown).

**Table 5:** Univariable odds ratios for association between 10-year cardiovascular risk and prescription of lipid lowering drugs.

Univariable Model	Patients (n = 365,718)		
	OR	95% CI	P value
10 year Cardiovascular Risk:			
<5%	1		<0.001
5%–9.9%	2.62	2.53–2.71	
10%–14.9%	4.32	4.17–4.47	
15%–19.9%	6.10	5.88–6.34	
20%–24.9%	7.73	7.42–8.06	
25%–29.9%	8.83	8.41–9.28	
≥30%	10.22	9.69–10.78	

*Based on analysis of 365,718 complete cases. Adjusted for clustering by practice.*

The analysis was repeated including all 1,364,383 patients. In this analysis, 27.9% of those eligible and 2.8% of those ineligible were started on treatment and only 38.2% of those prescribed lipid lowering drugs were eligible. In multivariable analysis the same factors predicted prescribing of lipid lowering drugs and odds ratios for predictors were very similar to those found with the complete case analysis. The strongest predictors were: increasing age (OR for age ≥65 years 4.27; 95% CI 4.12–4.43); diabetes (OR 4.81; 95% CI 4.69–4.93); total cholesterol level ≥7 mmol/L (OR 2.44; 95% CI 2.36–2.53) and ≥4 blood pressure measurements in the past year (OR for 4.29; 95% CI 4.12–4.46). In the analysis including patients with missing data the predictors of prescribing were similar in eligible and ineligible patients.

Patients with missing blood pressure or cholesterol measurements were much less likely to be prescribed lipid lowering drugs.

## Discussion

### Summary of main findings

Over half of patients without cardiovascular disease who were started on lipid lowering therapy were ineligible for treatment. Many eligible patients were not started on treatment. Most ineligible patients who were started on treatment were aged ≥55 years but not at high risk of CVD. Eligible patients who were non-diabetic and those with infrequent blood pressure measurements were unlikely to be started on treatment. The frequency of opportunities to prescribe appears to influence prescribing. We found no evidence of inequitable prescribing, as patients in deprived areas were slightly more likely to be prescribed lipid lowering drugs. However this finding should be treated with caution as deprivation was assessed by postcode of residence and allocated to quintiles.

We found evidence that GP prescribing is systematically influenced by cardiovascular risk factors – most strongly by older age, diabetic status and a total cholesterol level≥7 mmol/L. There was no evidence of a threshold effect at 20% ten-year CVD risk. Frequent blood pressure measurements (a proxy for cardiovascular related contacts) were also associated with treatment. Although all guidelines recommend treatment above a risk threshold and there is universal access to electronic risk calculators in UK primary care, cardiovascular risk was not the main predictor of prescribing. The patient characteristics associated with prescribing were similar in eligible and ineligible patients.

### Strengths and limitations of the study

The analysis uses a large dataset of electronic primary care records from across the whole of the UK and is representative of usual clinical care in measurement and recording of risk factors. We determined predictors of physician rather than patient behaviour as we are unable to identify whether prescribed drugs were collected or taken.

Absolute contraindications to lipid lowering drugs are uncommon and are unlikely to influence findings. We have no information on patients' treatment preferences, which are not predictable from patients' age, sex or risk factor status and may not accord with guideline recommendations [43], [44]. However there is little evidence that general practitioners take account of patients' preferences when starting preventive treatments [45].

### Comparison with existing literature

Our findings concur with previous studies reporting underuse of statins in primary care and greater prescribing of statins in patients with more risk factors [46], [47]. We confirmed that total cholesterol level and family history of premature coronary heart disease are predictors of statin prescribing [33], [48], [49]. We found no evidence of socioeconomic inequity in prescribing. There is little gradient in statin use across UK civil servants of different grades who were eligible for treatment [50]. Others found higher statin prescribing in more deprived UK communities [51].

We found diabetes to be a strong predictor of prescribing. Case vignette studies have demonstrated that both UK and Australian GPs are more likely to prescribe statins to eligible diabetics than eligible non-diabetics [52], [53].

LDL cholesterol levels above a threshold have been found to be an important of treatment in eligible patients [54]. Raised LDL cholesterol also predicts treatment in ineligible patients [55]. These echo our findings that total cholesterol levels ≥7.0 mmol/L were important predictors of prescribing.

Our finding of a relationship between prescribing and frequency of consultation is consistent with clinical inertia, a tendency to delay the decision to prescribe until the next visit [49], [56]. As patients aged 30 to 74 consult on average 5.6 times per year it is likely that over two years, GPs would have an opportunity to prescribe to the great majority of patients [57]. We also confirmed a link between antihypertensive prescribing and statin prescribing [49], [56].

We found that most statins are prescribed to patients who are not eligible for treatment. Overtreatment with statins has been reported from the USA, with a majority of those on treatment not meeting eligible under guidelines [58]. A study at a similar time reported overuse of statins in Norway [49]. However guidelines have changed substantially since this time. More recently, overuse of statins has been reported in the Netherlands, where a study reported that most patients on statins for primary prevention were not eligible and in Spain about one third were ineligible [49], [59], [60].

Overall we find more evidence to support the view that prescribing of statins is influenced more by single risk factors treated as categories (age ≥65 years; diabetes; total cholesterol ≥7 mmol/L) and frequency of contact with a clinician than by calculated CVD risk. The result is that there is a poor match between eligibility for lipid lowering treatment and being prescribed it.

Previous studies have shown variation in adherence to guidance in routine clinical practice [61]. Guidelines for assessment and follow up may be impractical [62]. Addressing the patient's primary concern may be a higher priority than prevention [63]. Clinicians and their patients may judge the costs and benefits of treatment differently to guideline authors. The Framingham equation overestimates the risk in populations with low CVD rates, which could justify lower use of statins [64]. Degree of adherence to guidelines may vary by health care centres [65]. Physicians who trained more recently are more likely to be guideline adherent [66]. As our anonymised data includes no information on general practitioner characteristics, we are unable to investigate the relationship between physician characteristics and prescribing.

The cost and cost-effectiveness implications of divergence from statins guidelines may be substantial [67]. Improving guideline compliance therefore has considerable potential to improve the cost effectiveness of prevention.

### Implication for future research

We should investigate whether poor discrimination in prescribing lipid lowering drugs extends to secondary prevention and to antihypertensive prescribing. Our findings are adjusted for the effects of practice, but the role of practice and GP characteristics on guideline adherence requires further analysis. While analysis of this kind can identify the importance of patient characteristics in influencing prescribing behaviour, it does not explain why or how these patient characteristics exert an influence. Divergence between prescribing behaviour and guidelines may reflect GPs' considered views about the effectiveness or adverse effects of treatment in relation to specific patient characteristics (e.g. prescribing to diabetics under the age of 40 years). If so these beliefs should be identified and tested against empirical evidence. If divergence between GP prescribing behaviour and guidelines may reflects lack of awareness of existing evidence (e.g. that lipid lowering drugs are effective in women), this can be addressed through better research dissemination. There is now strong evidence for the effectiveness of statins in primary prevention [68]. If the problem is mainly practical – (e.g. GPs only remembering to consider lipid lowering drugs in patients on antihypertensive treatment or having their blood pressure measured) the solutions may be practical steps such as electronic reminders. This analysis is therefore a first step in understanding why evidence based clinical guidelines do not translate into prescribing behaviour and represents a model for investigating the prescribing impact of other clinical guidelines.
